# Tenascin-c mediated vasculogenic mimicry formation via regulation of MMP2/MMP9 in glioma

**DOI:** 10.1038/s41419-019-2102-3

**Published:** 2019-11-21

**Authors:** Hai-ping Cai, Jing Wang, Shao-yan Xi, Xiang-rong Ni, Yin-sheng Chen, Yan-jiao Yu, Zi-wen Cen, Zhi-hui Yu, Fu-rong Chen, Cheng-cheng Guo, Ji Zhang, Chao Ke, Jian Wang, Zhong-ping Chen

**Affiliations:** 1 Department of Neurosurgery/Neuro-oncology, Sun Yat-sen University Cancer Center; State Key Laboratory of Oncology in South China; Collaborative Innovation Center for Cancer Medicine, Guangzhou, Guangdong 510060 P.R. China; 2 Department of Pathology, Sun Yat-sen University Cancer Center; State Key Laboratory of Oncology in South China; Collaborative Innovation Center for Cancer Medicine, Guangzhou, Guangdong 510060 P.R. China

**Keywords:** Cancer microenvironment, Tumour angiogenesis, Biological metamorphosis

## Abstract

Vasculogenic mimicry (VM), the formation of vessel-like structures by highly invasive tumor cells, has been considered one of several mechanisms responsible for the failure of anti-angiogenesis therapy in glioma patients. Therefore, inhibiting VM formation might be an effective therapeutic method to antagonize the angiogenesis resistance. This study aimed to show that an extracellular protein called Tenascin-c (TNC) is involved in VM formation and that TNC knockdown inhibits VM in glioma. TNC was upregulated with an increase in glioma grade. TNC and VM formation are potential independent predictors of survival of glioma patients. TNC upregulation was correlated with VM formation, and exogenous TNC stimulated VM formation. Furthermore, TNC knockdown significantly suppressed VM formation and proliferation in glioma cells in vitro and in vivo, with a reduction in cellular invasiveness and migration. Mechanistically, TNC knockdown decreased Akt phosphorylation at Ser^473^ and Thr^308^ and subsequently downregulated matrix metalloproteinase 2 and 9, both of which are important proteins associated with VM formation and migration. Our results indicate that TNC plays an important role in VM formation in glioma, suggesting that TNC is a potential therapeutic target for anti-angiogenesis therapy for glioma.

## Introduction

Glioma is the most common primary malignancy of the central nervous system. According to the WHO classification, gliomas are classified into four grades (grades I–IV), grades III and IV glioma are high-grade gliomas with poor prognosis. Excessive angiogenesis and adequate blood supply result in rapid proliferation and invasion in high-grade gliomas. Therefore, targeting angiogenesis may yield efficient anti-glioma therapies^1^. Receptor tyrosine kinase inhibitors (RTKIs) and recombinant humanized monoclonal antibodies have been widely studied in various cancers^2^. Bevacizumab (trade name Avastin) slightly contributed to progression-free survival (PFS) but not overall survival (OS) in glioma patients^3^. Several preclinical studies speculate that vasculogenic mimicry (VM) contributes to anti-angiogenesis therapeutic resistance^4–6^.

Structural features of VM were first reported by Maniotis et al. in 1999 as a phenomenon wherein aggressive tumor cells mimic vascular endothelial cells to form embryonic vasculogenic networks enriched in extracellular matrix (ECM) components, through which blood cells can be transported^7^. VM occurs in numerous solid tumors, including melanoma, head and neck carcinoma, breast cancer, and hepatocellular carcinoma^8–12^. We previously reported VM in glioma in 2005^13^. Further studies reported that VM promotes tumor cell invasion and migration, predicting poor clinical outcomes^14,15^.

Dynamic interactions among tumor cells, stromal cells, and the ECM is critical for glioma progression. Overexpression of Laminin-411 correlated with shorter survival of GBM patients and its depletion increased survival of host animals^16^. Integrins involve in the activation of transforming growth factor-β and promote invasiveness, angiogenesis, and maintains cancer cell stemness^17^. Similarly, tenascin-c (TNC), an ECM protein, is overexpressed under pathological conditions, such as injury and inflammation and in tumors^18,19^. TNC supports tumor cell proliferation and migration and correlates with a shorter disease-free time in glioma^20^. It was reported that TNC promoted glioma cell invasion and inhibited tumor proliferation^21^. TNC promoted growth of human brain tumor-initiating cells by activating NOTCH signaling^22^. TNC plays a proangiogenic role in proliferative diabetic retinopathy^23^; however, it is unclear whether TNC is involved in VM formation in glioma. This study aimed to investigate the role of TNC in VM formation and the effect of TNC knockdown on VM in glioma.

## Results

### TNC was upregulated with an increase in the pathological grade and VM formation in glioma

**Fig. 1:**
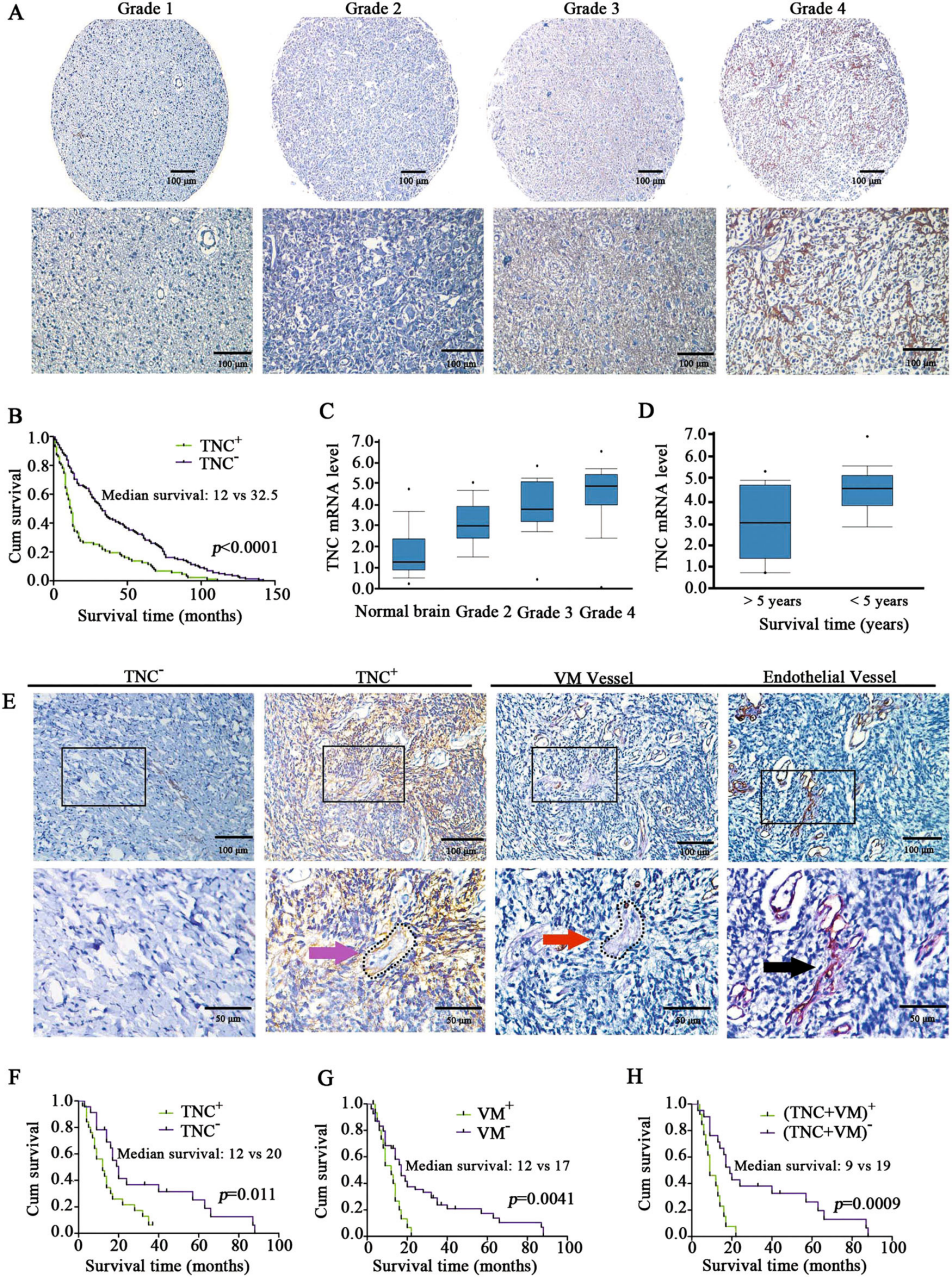
Tenascin-c (TNC) was upregulated with an increase in the pathological grade of glioma and vasculogenic mimicry (VM) formation. **a** Representative images of TNC expression in differently graded gliomas, as examined via immunohistochemistry. Scale bar = 100 μm. **b** Kaplan–Meier survival curve showing that TNC upregulation is correlated with poor prognosis in glioma patients (*p* < 0.001). **c**, **d** Analysis of ONCOMINE datasets show *TNC* mRNA upregulation in glioma (normal brain and grade II, III and IV; *n* = 23, 45, 31, and 81, respectively; Sun Brain Statistics dataset) and that patients with high TNC expression (*n* = 13) have a lower 5-year survival rate than those with low TNC expression (*n* = 413) (TCGA Brain Statistics dataset). **e** IHC results of VM vessels, endothelial cell-lined vessels, and TNC expression in patient-derived glioblastoma samples. The black arrows indicate endothelial cell-lined vessels; the red arrows indicate VM vessels (non-endothelial cell-lined and PAS-positive channels); TNC^−^ indicates TNC-negative glioma tissues; TNC^+^ indicates TNC-positive glioma tissues. Scale bar = 100 μm (upper), Scale bar = 50 μm (lower). **f**–**h** The Kaplan–Meier survival curves show that TNC upregulation and/or VM-positivity are associated with a poor prognosis (*p* < 0.05).

TNC expression was quantified in glioma cells via IHC staining on a tissue microarray containing 229 patient samples (12, 81, 72, and 64 cases of grade I, II, III, and IV glioma, respectively). TNC was significantly upregulated in high-grade glioma rather than in low-grade glioma (Fig. 1a and Supplementary Table 1) and was significantly correlated with a poor prognosis (*p* < 0.0001, Fig. 1b), concurrent with the results of the Sun Brain Statistics cohort in the ONCOMINE database showing the upregulation of *TNC* mRNA with an increase in glioma grade (Fig. 1c). Further, analysis of the association between *TNC* mRNA levels and patient prognosis based on a TCGA brain statistics dataset (*n* = 413) revealed that high *TNC* mRNA levels were associated with a poor prognosis compared to low *TNC* mRNA levels (Fig. 1d). Thereafter, we assessed TNC expression, VM vessels (CD31-negative, PAS-positive), and endothelial vessels (CD31-positive, PAS-positive) in 50 GBM samples. Images of negative TNC staining, positive TNC staining, and the typical morphology of VM (red arrow) and endothelial vessels (black arrow) are shown in Fig. 1e. Our results show that 34% samples (17/50, Table 1) were VM-positive, 54% samples (27/50, Table 2) were TNC-positive, and 88% (15/17) of VM-positive samples were TNC-positive (Fig. 1e, pink arrow, Table 1). Multivariate Cox regression analysis revealed that TNC expression was significantly correlated with VM formation (*χ*^2^ = 13.788, *p* = 0.001, Table 1) but not with age, tumor size, or sex (Table 2). Kaplan–Meier analysis indicated that GBM patients with increased TNC expression, VM positivity, or both had shorter survival times (*p* = 0.011, *p* = 0.0009, and *p* = 0.0041, respectively) than those without either or both of these features (Fig. 1f–h). These results indicate that TNC expression levels are correlated with the glioma grade and VM formation and potentially predict a poor prognosis in glioma patients.

**Table 1:** The correlation between vasculogenic mimicry formation with clinicopathological features in 50 glioblastoma samples.

	No.	VM		*χ*^2^	*p*-value
		Positive	Negative		
Sex					
Male	33	14	19	3.07	0.117^a^
Female	17	3	14		
Age (years)					
<50	27	8	19	0.5	0.557^a^
≥50	23	9	14		
Tumor size (diameter)					
<5	21	10	11	2.993	0.131^a^
≥5	29	7	22		
IDH					
Yes	7	3	4	0.011	0.9182^b^
No	43	14	29		
MGMT					
Yes	25	10	15	0.802	0.370^a^
No	25	7	18		
Preoperative epilepsy					
Yes	10	4	6	0.006	0.941^b^
No	40	13	27		
MVD					
<median	28	5	23	7.39	0.007^a^
>median	22	12	10		
Extent of tumor resection					
Gross total/subtotal	38	13	25	0.000	1.000^b^
Partial	12	4	8		
TNC					
Positive	27	15	12	13.788	0.001^a^
Negative	23	2	21		

^a^Pearson’s *χ*^2^ test (asymptotic significance, two-sided)

^b^Continuity correction (two-sided)

**Table 2:** The correlation between tenascin-c expression with clinicopathological features in 50 glioblastoma samples.

	No.	TNC		*χ*^2^	*p*-value
		Positive	Negative		
Sex					
Male	33	19	14	0.500	0.48^a^
Female	17	8	9		
Age					
<50	27	13	14	0.809	0.368^a^
≥50	23	14	9		
Tumor size (diameter)					
<5	21	10	11	0.593	0.441^a^
≥5	29	17	12		
IDH					
Yes	7	1	6	3.476	0.062^b^
No	43	26	17		
MGMT					
Yes	25	13	12	0.081	0.777^a^
No	25	14	11		
Preoperative epilepsy					
Yes	10	5	5	0.000	1.000^b^
No	40	22	18		
MVD					
<median	28	6	22	27.179	0.001^a^
>median	22	21	1		
Extent of tumor resection					
Gross total/subtotal	38	21	17	0.102	1.000^a^
Partial	12	6	6		
VM					
Positive	17	15	2	12.154	0.001^a^
Negative	33	12	21		

^a^Pearson’s *χ*^2^ test (asymptotic significance, two-sided)

^b^Continuity correction (two-sided)

### TNC expression was correlated with VM formation in vitro

**Fig. 2:**
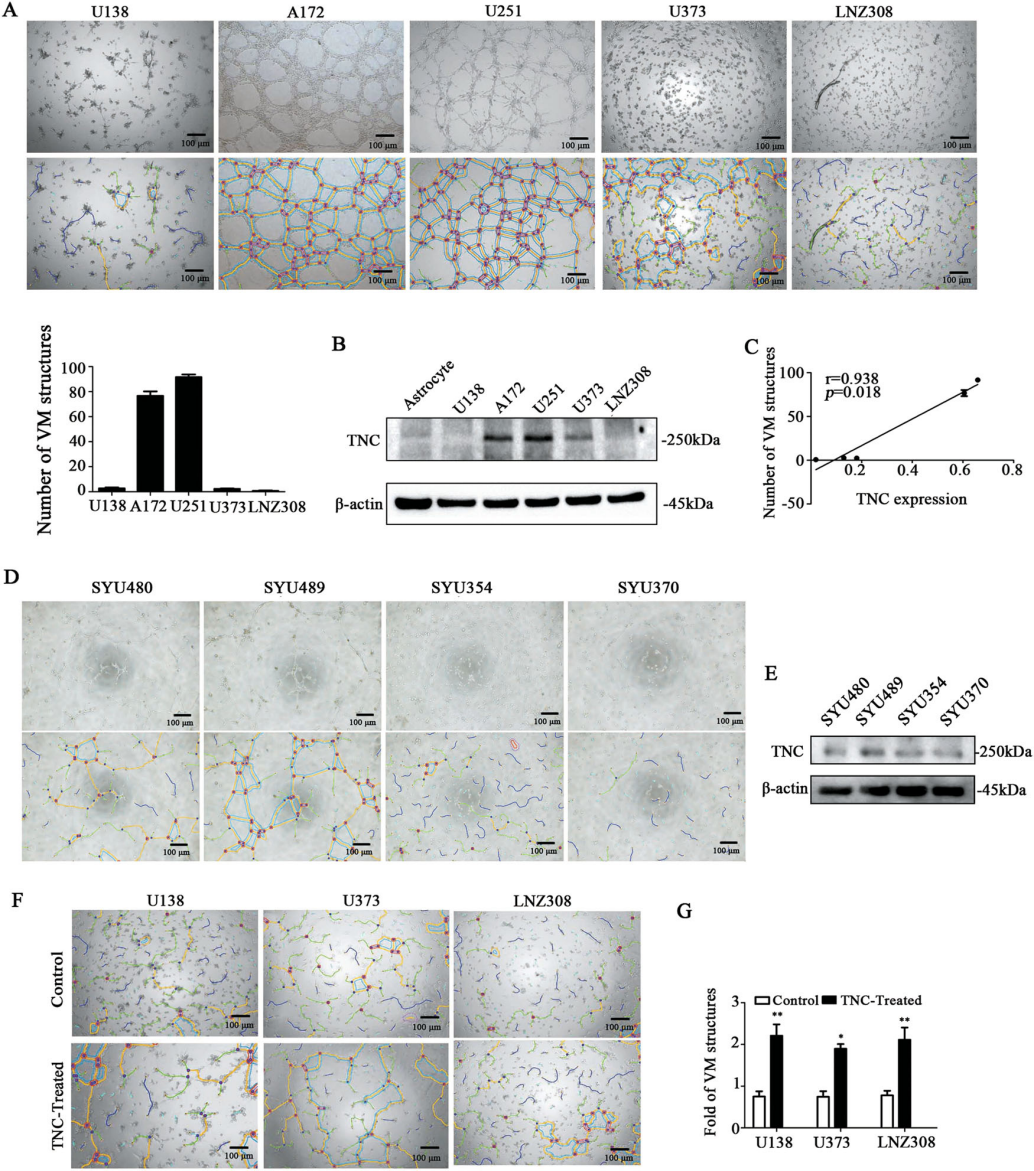
Tenascin-c (TNC) expression is correlated with vasculogenic mimicry (VM) formation in glioma cells. **a** Different potentials for VM formation in five glioma cell lines were assessed via 3D Matrigel culturing in vitro, which is a universal method for VM evaluation in vitro. Both U251 and A172 cells formed VM-like structures, while U373, U138, and LNZ308 cells did not (the blue mesh indicates VM-like structures). Scale bar = 100 μm. **b** Western blot analysis revealed that TNC was upregulated in U251 and A172 cells rather than in U373, U138 and LNZ308 glioma cell lines (β-actin used as the loading control). **c** Correlation analysis revealed that the number of VM structures was correlated with TNC protein expression (*r* = 0.938, *p* < 0.05). **d**, **e** VM formation ability was stronger in primary cultured cells with higher level of TNC than those with low level of TNC. Glioma cell SYU489 formed VM-like structures, while SYU480, SYU354, and SYU370 did not. At the meanwhile, the expression level of TNC was higher in SYU489 rather than in SYU480, SYU354, and SYU370. **f**, **g** Exogenous TNC (10 μg/ml) was used to stimulate U138, U373, and LNZ308 cells for 8 h, and the number of VM structures increased in comparison with that in the control groups. Scale bar = 100 μm. (*p < 0.05, **p < 0.01, ***p < 0.001).

To clarify the role of TNC in VM formation, we assessed VM formation in glioma cells in vitro. As shown in Fig. 2a, among the five glioma cell lines used herein, U251 and A172 cells formed typical closed circle-like VM structures (the blue mesh indicates VM structures), while U138, U373, and LNZ308 cells exclusively formed open branch-like structures. As expected, TNC was upregulated in U251 and A172 cells but downregulated in U138, U373, and LNZ308 cells (Fig. 2b), in accordance with the VM formation ability (*r* = 0.938, *p* < 0.05, Fig. 2c). The VM formation ability was also evaluated in four primary cultured low-passage glioma cells (less than 10 passages) derived from glioma patients. Glioma cells with high TNC expression formed more closed circle-like VM structures and vice versa (Fig. 2d, e). To further confirm the role of TNC in VM formation, TNC (10 μg/ml) was exogenously supplemented in suspensions of U138, U373, and LNZ308 cells, and the cells were subjected to three-dimensional culturing. Eight hours later, the VM formation was significantly greater in TNC-treated than in untreated cells (*p* < 0.05; Fig. 2f, g), suggesting that TNC is involved in VM formation in glioma cell lines.

### TNC knockdown attenuated VM formation in vitro and in vivo

**Fig. 3:**
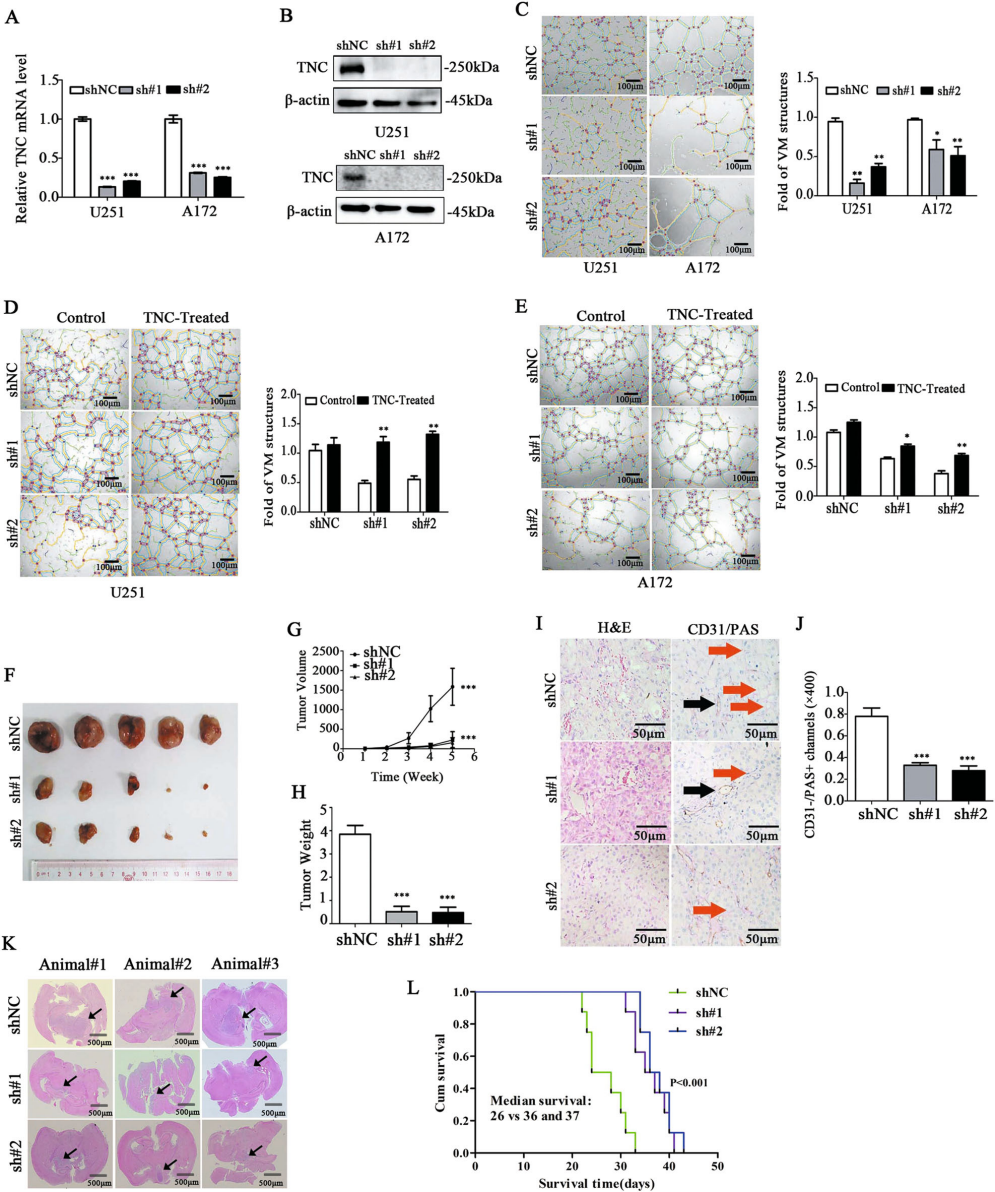
Tenascin-c (TNC) knockdown attenuated vasculogenic mimicry (VM) formation in vitro and in vivo. **a** Total RNA was isolated from the shNC, sh#1, and sh#2 groups of U251 and A172 cells and analyzed via RT-qPCR. *TNC* mRNA was significantly downregulated (*p* < 0.01; *GAPDH* used as the control). **b** Western blot analysis revealed that TNC was significantly downregulated (β-actin used as the loading control). **c** The number of VM structures in both the sh#1 and sh#2 groups of U251 and A172 glioma cells was decreased in comparison with that in the shNC group. Scale bar = 100 μm. **d**, **e** Exogenous TNC exposure for 8 h increased the number of VM structures in both the sh#1 and sh#2 groups of U251 and A172 glioma cells. Scale bar = 100 μm. **f**, **g** TNC knockdown inhibited tumorigenicity in U251 glioma cells. **h** Quantification of tumor mass in the shNC, sh#1, and sh#2 groups. **i** Representative images of hematoxylin-eosin staining and CD31/periodic acid–Schiff (PAS) staining (the red arrows indicate typical VM channels; the black arrows indicate classical endothelial cell vessels). **j** Quantification of VM channels via CD31/PAS staining in the shNC, sh#1, and sh#2 groups (magnification: ×400; scale bar = 50 μm). **k** HE staining of brain sections demonstrated a significant decrease in tumor volume after TNC-knockdown at 25 days post implantation (black arrows indicate tumor location). **l** Kaplan–Meier survival curves showing a significant increase in median survival of TNC-knockdown tumor-bearing mice. Scale bar = 500 μm (*p < 0.05, **p < 0.01, ***p < 0.001).

To determine the precise role of TNC in VM formation, we knocked down TNC in U251 and A172 cells with two specific shRNA sequences and control scramble shRNA. TNC was successfully knocked down in U251 and A172 cells (***p < 0.001, Fig. 3a, b), and VM was significantly reduced in these cells (*p < 0.05, **p < 0.01; Fig. 3c). Upon treatment of TNC-knockdown cells with exogenous TNC, VM formation normalized to baseline levels (*p < 0.05, **p < 0.01; Fig. 3d, e). Upon TNC knockdown, glioma growth was significantly inhibited in mice bearing subcutaneous xenograft of U251 cells (***p < 0.001, Fig. 3f–h). The orthotopic animal model also shown that those mice injected U251 cell after TNC knockdown survived longer. Histologic analysis of brain specimens from xenografts demonstrated a significant decrease in tumor volume after TNC-knockdown at 25 days post implantation (black arrows indicate tumor location) (***p < 0.001, Fig. 3k, l). Furthermore, VM (CD31^−^/PAS^+^, Red arrows) in U251 xenograft tissues was significantly decreased in sh#1 and sh#2 groups compared with that in the shNC group (***p < 0.001, Fig. 3i, j; Supplementary Fig. S1C, D). Therefore, our data support the hypothesis that TNC is involved in VM formation in glioma cells.

### TNC knockdown inhibited proliferation, invasion, migration, and induced apoptotic cell death in glioma cells

**Fig. 4:**
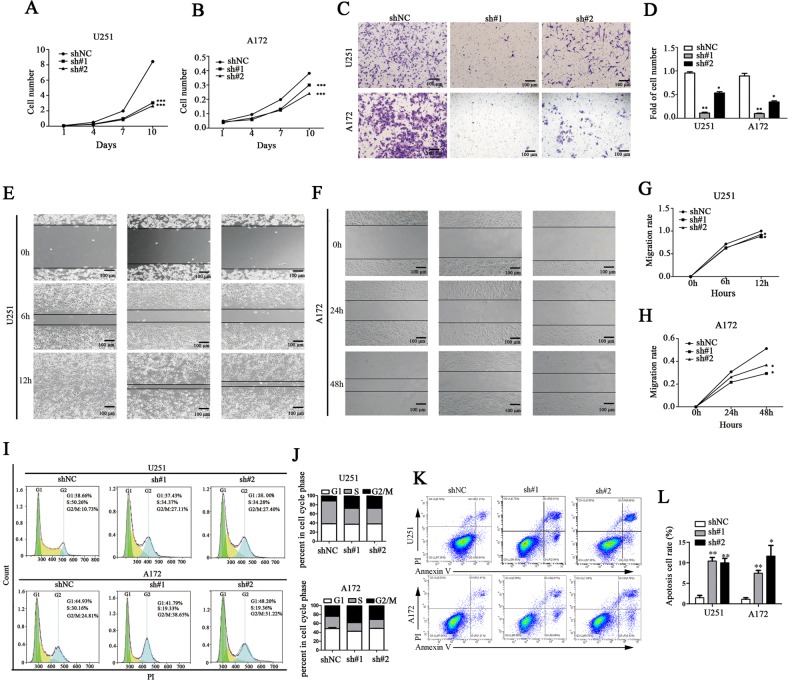
Tenascin-c (TNC) knockdown inhibited proliferation, invasion, migration, and induced apoptotic death in glioma cells. **a**, **b** The cell proliferation assay revealed that TNC knockdown (sh#1 and sh#2 groups) inhibited U251 and A172 glioma cell growth in vitro. **c**, **d** Glioma cell invasiveness was assessed via a Transwell assay. Cells penetrating 8.0-μm PET membranes coated with Matrigel. Representative images of each group of U251 and A172 cells indicated that the cellular invasiveness in the sh#1 and sh#2 groups was attenuated in comparison with that in the corresponding shNC groups. Scale bar = 100 μm. **e**–**h** Cellular migration in both the sh#1 and sh#2 groups of U251 and A172 cells was inhibited, as determined via a wound-healing assay. Scale bar = 100 μm. **i**, **j** TNC knockdown induced G2/M arrest in both U251 and A172 cells. **k**, **l** TNC-knockdown increased apoptotic cells rate in both U251 and A172 glioma cells. (*p < 0.05, **p < 0.01, ***p < 0.001).

VM formation is closely associated with tumor cell proliferation, invasion, and migration^24^. Thus, we investigated the detailed role of TNC in glioma cells. From the 4th passage proliferation was significantly lower in TNC-knockdown U251 and A172 cells than in control cells (***p < 0.001, Fig. 4a, b). Furthermore, TNC knockdown induced G2/M arrest in both U251 and A172 cells (Fig. 4i, j). Transwell and wound-healing assays to investigate the effect of TNC on cell invasion and migration revealed that a significant reduction in both cell invasion and migration upon TNC knockdown (*p < 0.05, **p < 0.01; Fig. 4c, d, Supplementary Fig. S1A, B). Furthermore, the rate of wound healing was significantly decreased in TNC-knockdown cells (*p < 0.05, Fig. 4e–h). Given that stromal-derived TNC increases metastasis by reducing apoptosis and inducing the cellular plasticity of cancer cells^25^, we then investigated whether TNC-knockdown induced apoptosis in glioma cell. As expected, TNC-knockdown increased Annexin V positive cells in both U251 and A172 glioma cells (*p < 0.05, **p < 0.01; Fig. 4k, l). These results indicated that TNC is involved in cell proliferation, cell cycle progression, invasion, and migration in glioma cells.

### TNC knockdown inhibited Akt phosphorylation and downregulated MMP2/9

**Fig. 5:**
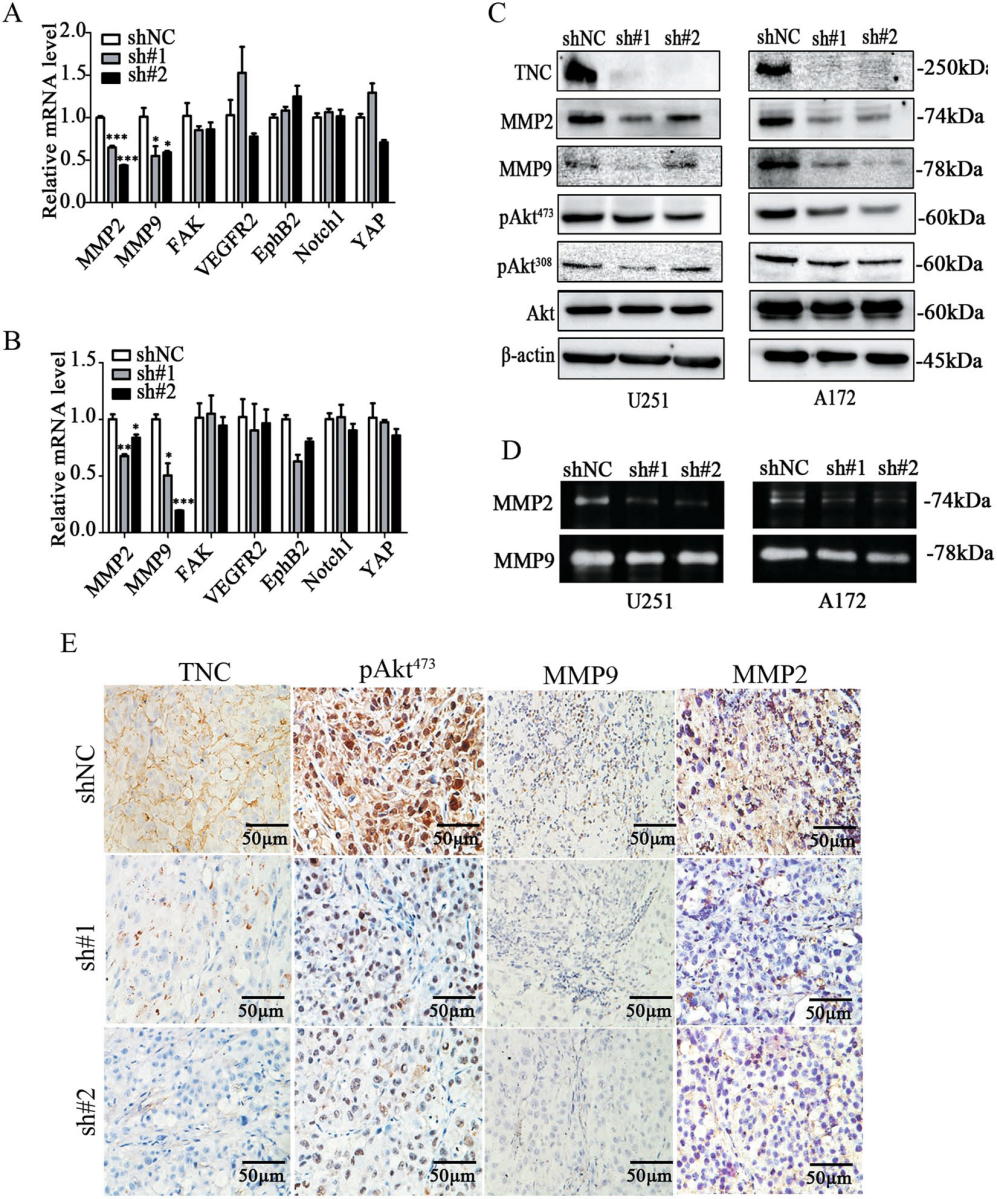
Tenascin-c (TNC) knockdown inhibited Akt phosphorylation and downregulated matrix metalloproteinase (MMP) 2/9. **a**, **b** Total RNA was isolated from the shNC, sh#1, and sh#2 groups of U251 and A172 cells. The mRNA levels of VM-related markers were analyzed via RT-qPCR (*GAPDH* used as the internal control). *MMP2* and *MMP9* mRNA were significantly downregulated. **c** Akt phosphorylation at Ser^473^ and Thr^308^ residues and MMP2 and MMP9 expression were decreased after TNC knockdown (β-actin used as the control). **d** MMP2/9 activity was inhibited, as confirmed via zymography. **e** TNC, pAKT, MMP2, and MMP9 downregulation in tumor tissues from xenografts with TNC knockdown (magnification: ×400; scale bar = 50 μm). (*p < 0.05, **p < 0.01, ***p < 0.001).

VM formation involves numerous factors including VE-cadherin, Twist, VEGFR2, Akt, and MMP2/9^26–28^. Herein, *MMP2* and *MMP9* mRNA (primer sequence described in Supplementary Table 2) were significantly downregulated in TNC-knockdown cells (Fig. 5a, b). Since MMP2 and MMP9 are important downstream effectors of Akt, herein, TNC knockdown impaired Akt phosphorylation at both Ser^473^ and Thr^308^ and downregulated MMP2 and MMP9 (Fig. 5c). Gelatin zymography confirmed that MMP2 and MMP9 activity were reduced in culture supernatants in TNC-knockdown cells (Fig. 5d). IHC analysis of U251 subcutaneous and intracranial xenografts tissues revealed that Akt phosphorylation at Ser^473^ and MMP2 and MMP9 expression were decreased (Fig. 5e and Supplementary Fig. S1E). These data show that TNC knockdown decreases Akt phosphorylation and MMP2/9 activity.

### MK-2206 treatment downregulated MMP2 and MMP9 and inhibited VM formation

**Fig. 6:**
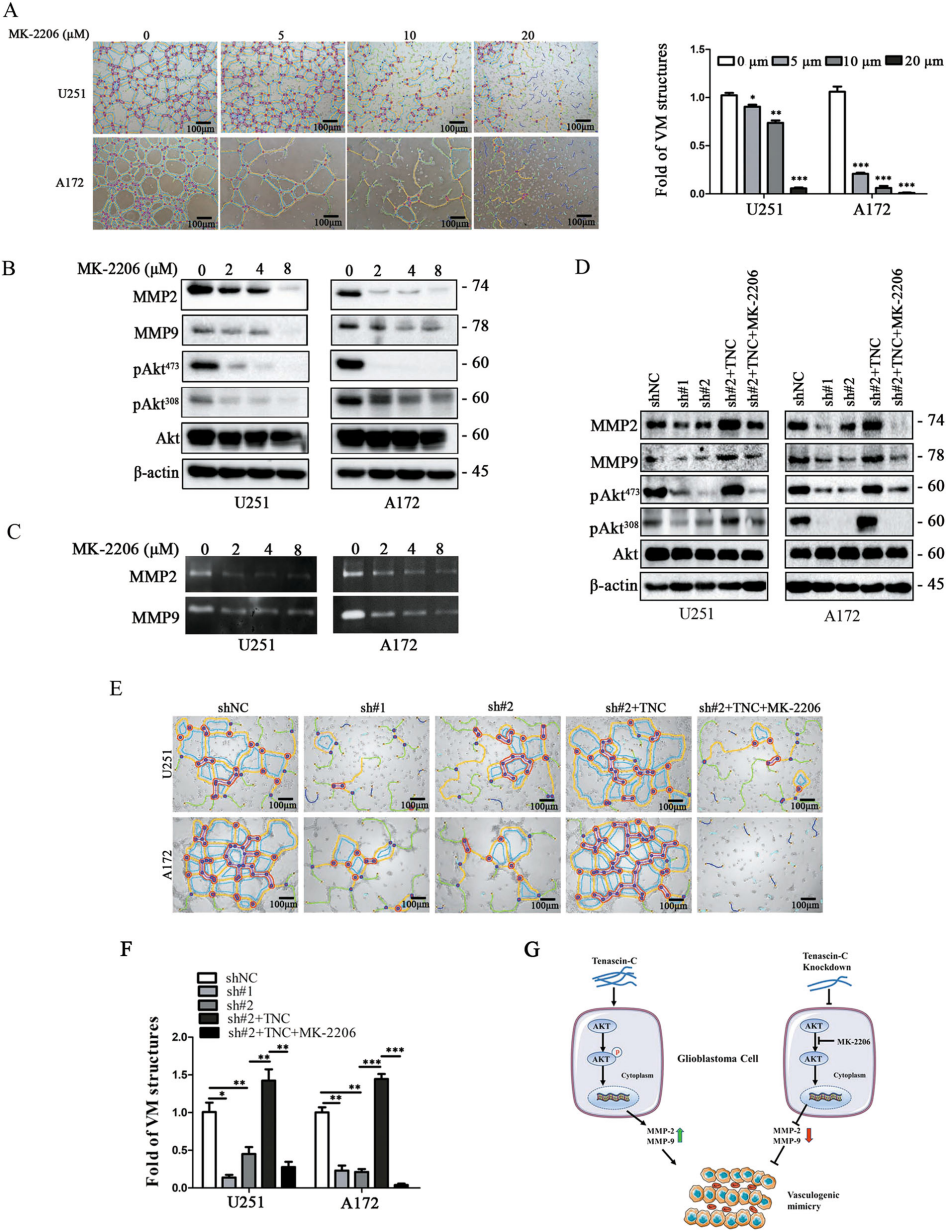
MK-2206 treatment downregulated matrix metalloproteinase (MMP) 2/9 and inhibited vasculogenic mimicry (VM) formation. **a** VM formation was assessed in U251 and A172 cells after exposure to the Akt phosphorylation inhibitor MK-2206 at 0, 5, 10, and 20 μM for 24 h. The number of VM structures decreased in a dose-dependent manner. (magnification: ×100; scale bar = 100 μm). **b** Immunoblotting revealed that MMP2/9 expression and Akt phosphorylation at both Ser^473^ and Thr^308^ residues were significantly downregulated in a dose-dependent manner after MK-2206 treatment. **c** Zymography revealed that MMP2 and MMP9 activity was inhibited after MK-2206 treatment. **d** Immunoblotting revealed that MMP2/9 expression and Akt phosphorylation at both Ser^473^ and Thr^308^ residues were increased after TNC treatment for 24 h and decreased upon combinatorial treatment with TNC and 10 μM MK-2206. **e**, **f** The VM formation assay revealed that VM structures increased after TNC treatment for 24 h and decreased upon combinatorial treatment with TNC and 10 μM MK-2206. **g** A model describing the mechanism of how TNC involved the regulation of VM formation process. (*p < 0.05, **p < 0.01, ***p < 0.001).

To determine that Akt phosphorylation mediates MMP2/MMP9 expression and VM formation, MK-2206, a highly selective small molecular inhibitor blocks the phosphorylation of Akt1, Akt2, and Akt3, was used. As expected, VM was reduced gradually in a dose-dependent manner in U251 and A172 cells upon exposure to MK-2206 at 0, 5, 10, and 20 μM for 24 h (Fig. 6a). Furthermore, Akt phosphorylation at Ser^473^ and Thr^308^ residues and MMP2 and MMP9 were downregulated in cells treated with MK-2206 at 2, 4, and 8 μM for 24 h (Fig. 6b). Gelatin zymography after MK-2206 treatment indicated are duction in MMP2 and MMP9 activity (Fig. 6c). A rescue assay revealed that both Akt phosphorylation at Ser^473^ and Thr^308^ residues and MMP2 and MMP9 expression levels in the sh#2 group were increased upon treatment with exogenous TNC and decreased upon treatment with MK-2206 at 10 μM for 24 h (Fig. 6d). As expected, the VM formation assay revealed consistent results with the rescue assay (Fig. 6e, f). These data show that MK-2206 treatment decreased Akt phosphorylation and MMP2/9 activity, further reducing VM formation, indicating that the Akt/MMP2/MMP9 axis is involved in TNC-regulated VM formation.

## Discussion

High-grade glioma is characterized by a highly vascularized tumor. Anti-angiogenesis therapy might be a new method for high-grade glioma patients since Folkman et al. proposed the tumor angiogenesis theory in 1971^1^. Common genetic alterations in GBM like amplification of the epidermal growth factor receptor (EGFR) and its mutant EGFRVIII promote angiogenesis in GBM implying an important role of endothelial vessels in GBM development^29^. However, tumor cell-derived patterns, such as VM^30^, potentially play a vital role in tumorigenesis and contribute to anti-angiogenesis therapeutic resistance. Bevacizumab abrogates endothelial cell-driven angiogenesis but not VM formation^31^. The mechanism underlying VM formation is unclear, although it was proposed by Maniotis et al. in 1999^7^. Our finding that TNC promotes VM formation by glioma cells further indicates an important role of TNC in glioma pathogenesis.

The present results confirmed TNC upregulation in glioma tissues, especially in GBM tissues and show that 34% of samples were VM-positive, as reported previously^32^. We assessed VM formation in glioma cell lines and low-passaged primary cultured glioma cells, and found that TNC expression levels correlated with VM formation ability. Exogenous TNC promoted VM formation by glioma cells, thus indicating a direct interaction between TNC and VM formation. Furthermore, TNC knockdown decreased VM formation in vitro and in vivo. Tumor cell proliferation, invasiveness, and migration are critical for VM formation;^33^ concurrently, cell growth, invasion, and migration were inhibited upon TNC knockdown. Furthermore, TNC knockdown significantly induced G2/M arrest and apoptotic cell death in glioma cells. Assessment of VM markers in U251 and A172 cells after TNC knockdown revealed that MMP2 and MMP9 mRNA were significantly downregulated. MMP2 and MMP9 are important downstream effectors of Akt and contribute to VM formation^24^. We investigated whether TNC impaired VM formation in glioma cells upon a reduction in Akt phosphorylation and MMP2/9 downregulation. Indeed, TNC knockdown decreased Akt phosphorylation at Ser^473^ and Thr^308^ residues and downregulated MMP2/9. Furthermore, MK-2206 was used to inhibit Akt phosphorylation at both Ser^473^ and Thr^308^ residues; consequently, VM decreased in a dose-dependent manner. We demonstrated that TNC promoted VM formation by activating Akt phosphorylation and inducing MMP2/MMP9 expression. Left panel, Extracellular protein TNC activates Akt phosphorylation and then stimulates the expression of MMP2/MMP9, which further promotes VM formation. Right panel, TNC-knockdown decreases Akt phosphorylation and MMP2/MMP9 expression, as well as downstream VM formation. Similarly, small molecular inhibitor MK-2206 attenuates Akt phosphorylation, which decreases expression of MMP2/MMP9 and VM formation (Fig. 6g). These results suggest that Akt phosphorylation and MMP2/9 expression play an important role in TNC-induced VM formation.

In conclusion, our results show that TNC promotes VM formation in glioma, thus potentially contributing to anti-angiogenic therapeutic resistance. Our results show that an Akt/MMP2/MMP9 axis potentially regulates VM formation in glioma. Therefore, targeting TNC expression is a potentially useful method to inhibit VM formation in glioma and decrease anti-angiogenic therapeutic resistance.

## Materials and methods

### Patients and cell lines

Fifty glioblastoma (GBM) specimens and a tissue array (229 glioma samples) were obtained from patients who received surgery at Sun Yat-sen University Cancer Center (SYSUCC) between 2001 and 2016 with written informed consent. General information of the cohorts is summarized in Tables 1, 2 and Supplementary Table 1. Tumors were pathologically diagnosed by experienced pathologists. Overall survival (OS) of the patients was defined as the period from the day of surgery to death. The latest follow-up data were updated on December 31, 2017. Experiments were conducted in accordance with the guidelines and approved by the Ethics Committee of SYSUCC.

Cell line U251 and U373 were maintained from the State Key Laboratory of Oncology in South China. A172, U138, LNZ308 and normal astrocyte cell line (Ast) were obtained from Dr. Shing-shun Tony To, Department of Health Technology and Informatics, The Hong Kong Polytechnic University. All these cell lines were authenticated by STR profiling within 6 months. Astrocytes were cultured in astrocyte medium (ScienCell Research Laboratories, Carlsbad, CA,) supplemented with fetal bovine serum, astrocyte growth supplement, and penicillin/streptomycin. Purified human Tenascin-c protein was purchased from EMD/Millipore (Darmstadt, Germany).

### Tumor specimens and primary cell culture

Four primary high-grade glioma specimens were obtained freshly from the operating room following protocols approved by the research ethics committee in the Sun Yat-Sen University Cancer Center with informed consent obtained from all subjects. Primary glioma cell lines named (SYU480, anaplastic astrocytoma; SYU489, glioblastoma; SYU354, glioblastoma; SYU370, anaplastic astrocytoma) were isolated and subsequently cultured in DMEM supplemented with 10% fetal bovine serum and penicillin/streptomycin as reported by Jun Fu et al.^34^.

### Immunohistochemical (IHC) staining

IHC staining was performed as reported previously^35^. The expression level of TNC in glioma tissues was scored as the proportion of the area with positive staining (0–100%) multiplied by the staining intensity (0, negative; 1, weak; 2, moderate; 3, intense). The scores were determined by two pathologists independently. The median score was chosen as the cut-off value for defining high and low expression. Sections were probed with primary anti-TNC (ab108930, Abcam, Cambridge, MA), anti-CD31 (ab134168, Abcam), anti-pAkt (4060P, Cell Signaling Technologies [CST], Danvers, MA,), anti-matrix metalloproteinase (MMP) 2 (ab86607, CST), and anti-MMP9 (ab76003, Abcam) antibodies overnight at 4 °C, and the antibodies were detected using the DAB system (Golden Bridge, Beijing, China).

### TNC knockdown

TNC was silenced in U251 and A172 glioma cells, using shRNA vectors based on the pLKO.1 plasmid (#10879, Addgene, Watertown, MA). Two sequences targeting human TNC, along with a negative control sequence, were used. Lipofectamine 3000 (Invitrogen, Carlsbad, CA) was used for transfection in accordance with the manufacturer’s protocol. Transfected cells were cultured in selection media supplemented with 2 μg/ml puromycin (MCE, Monmouth Junction, NJ) for 2 weeks.

### Proliferation assay

The role of TNC in glioma cell proliferation was evaluated via cell growth assays, as reported previously^35^. Briefly, 5000 cells were seeded in each well in 6-well plates. The cell number was determined using a Celigo system every 3 d (Nexcelom Bioscience LLC, Lawrence, MA). Each cell line was plated in triplicate, and the experiments were repeated at least three times.

### Cell cycle analysis

Cells were harvested, fixed with 70% ethanol overnight at 4 °C, and stained with propidium iodide (KeyGEN, Jiangsu, China) in the dark for 1 h. Cell suspensions were subjected to flow cytometry with an ACEA NovoCyte system (ACEA Biosciences, Inc., Santa Clara, CA). Cell cycle distribution was analyzed with 100,000 events for each sample.

### Flow cytometry analysis

Flow cytometry analysis was used to detected apoptotic cell death. Briefly, cell suspensions were blocked with 10% BSA for 10 min. Apoptosis were detected using Annexin V–FITC/PI detection kit (BD biosciences, New Jersey, USA). Flow cytometry analysis was performed using a CytoFLEX (Beckman Coulter Inc., CA, USA) flow cytometer equipped with CytExpert software with 20,000 events recorded for each sample.

### Cell invasion and migration assays

Serum-free cell suspensions containing 5 × 10^4^ cells were seeded in the upper chamber coated with or without Matrigel for invasion or migration assays, respectively. DMEM medium supplemented with 10% fetal bovine serum was added to the bottom chamber. After incubation at 37 °C for 24 h, the migrated cells were fixed in 10% formalin and stained with 0.4% crystal violet. Images were captured in five random fields, and cells were enumerated using a bright-field microscope (100×). Each experiment was performed independently in triplicate.

### Wound healing assay

Cells were cultured in 6-well plates up to 80–90% confluence. An even scratch was made in the middle of each well, using a 10-μl pipette tip, and cells were then washed with PBS and incubated in serum-free medium at 37 °C for 48 h. Images of the wounds were captured every 6 h, and the wound closure ratio was determined as the ratio of the distance of cell migration to the width of the wound at 0 h until the wounds were closed.

### Quantitative real-time PCR analysis

Total RNA was extracted with TRIzol (Invitrogen) and reverse-transcribed to cDNA using a cDNA Synthesis kit (Invitrogen) in accordance with the manufacturer’s protocol. Real-time quantitative PCR (qRT-PCR) was subsequently performed using a Bio-Rad CFX96 Real-Time PCR System (Bio-Rad Laboratories, Inc., Hercules, CA). All samples were analyzed in triplicate, and mRNA expression was normalized to that of the housekeeping gene *GAPDH*, using the 2^−ΔΔCt^ method.

### Three-dimensional culturing

VM formation ability in vitro was assessed via three-dimensional culturing. Briefly, 96-well plates were coated with 50 μl of Matrigel (BD Biosciences, Sparks, MD), and cell suspensions were seeded on Matrigel and incubated for 8 h at 37 °C. Five random fields per well were imaged using a bright-field microscope (magnification, ×100). The experiment was performed in triplicate. The total number of cells on the mesh was determined and analyzed using ImageJ software (National Institutes of Health, Bethesda, MD).

### Western blot analysis

Western blot analysis was performed using a standard protocol^36^. Briefly, equal amounts of protein (30 μg) were separated via SDS-PAGE and electro-transferred onto polyvinylidene difluoride membranes (EMD Millipore). Membranes were probed with primary anti-TNC (ab108930, Abcam), anti-MMP2 (ab86607, Abcam), anti-MMP9 (ab76003, Abcam), anti-pan-Akt (4691 s, CST), anti-pAkt^473^ (4060 P, CST), anti-pAkt^308^ (sc-135650, Santa Cruz Biotechnology, Santa Cruz, CA), anti-β-actin (8457 S, CST) antibodies and then probed sequentially with secondary goat anti-mouse and anti-rabbit antibodies (7076P2, 7074P2, CST) and visualized using an enhanced chemiluminescence kit (Beyotime, Shanghai, China).

### Gelatin zymography

Total proteins were extracted from culture supernatants and separated via PAGE on 7.5% polyacrylamide gels containing 0.5% gelatin (Macklin, Shanghai, China). Gels were then equilibrated with 2.5% Triton X-100 and incubated in substrate buffer (50 mmol/L Tris-HCl [pH 7.5], 150 mmol/L NaCl, and 10 mmol/L CaCl_2_) for 24 h at 37 °C. Gels were stained with Coomassie Brilliant Blue G250 (BioFROXX, Guangzhou, China) for 2 h and washed until clear zones associated with MMP activity were observed.

### Akt inhibition via MK-2206 treatment

Cells were treated with the small-molecule Akt inhibitor MK-2206 (Selleckchem, Houston, TX) for 24 h at 5, 10, and 20 μM for the VM formation assay and 2, 4, and 8 μM for Western blot analysis. Cells and culture supernatants were then harvested for subsequent analyses.

### Xenograft animal model

Mice were allocated to experimental groups randomized before the implantation of tumor cells. For the subcutaneous animal model, U251 cells at a density of 10^7^ cells were injected subcutaneously into the flanks of 4–6-week-old female BALB/c nude mice (*n* = 15) (Model Animal Research Center of Nanjing University, Nanjing, China). Tumor volumes were monitored every 3 d, and tumor sizes were calculated using the following formula: tumor volume = 0.5 × length × (width)^2^. Five weeks later, the mice were euthanized, and tumors were dissected out for further IHC analysis.

For the orthotopic animal model, U251 cells resuspended in PBS were intracranially injected 1 mm lateral and 2 mm posterior to bregma and 4 mm deep to the surface of the skull using a micro-syringe and 27 G needle as in our previous study^34^ [*n* = 13/group, 2.0 × 10^5^ cells/mouse]. Mice (*n* = 8) were observed every 2 day until moribund and then sacrificed. Another cohort (*n* = 5) was sacrificed 25 days post implantation. Brains were removed, embedded in paraffin, and sectioned for HE staining. All animal experiments were performed in accordance with institutional guidelines and approved by the Animal Care and Use Ethical Committee of SYSUCC.

### Statistical analyses

Data were analyzed using the SPSS software (Version 17.0, Chicago, IL, USA) and are presented as the mean ± standard deviation (S.D.) or standard error (S.E.M.). Statistical significance was determined at a *p*-value of < 0.05. Pearson’s correlation analysis was performed to determine the correlation between VM and TNC expression. Differences were compared using Student’s *t*-test for two groups and one-way ANOVA for multiple groups. Survival probabilities were plotted using the Kaplan–Meier method.

## Supplementary information

Supplemental information

Supplementary Figure 1
