# Supplementary material for: Tenascin-c mediated vasculogenic mimicry formation via regulation of MMP2/MMP9 in glioma
Source: Cell Death Dis. 2019 Nov 21;10(12):879. doi: 10.1038/s41419-019-2102-3 (PMC6872754; doi:10.1038/s41419-019-2102-3)
Supplement: Supplementary file 1 — Supplemental information [file 41419_2019_2102_MOESM1_ESM.docx]

Supplementary Information

**Supplementary Figure Legends**

**Supplementary Figure 1. TNC-knockdown inhibited migration, VM formation, Akt phosphorylation and downregulated MMP 2/9 expression in glioma cell. (**A，B) The migratability of U251 and A172 cells in the knockdown groups was attenuated in comparison with that in the corresponding shNC groups. (C) Quantification of VM channels in tumor from brain specimens of mice via CD31/PAS staining in the shNC, sh#1, and sh#2 groups (magnification: 400×; scale bar=50μm). (D) Representative images of HE staining and CD31/ PAS staining in tumor from brain specimens of mice (the red arrows indicated typical VM channels; the black arrows indicated classical endothelial cell vessels). (E) TNC, pAKT, MMP2, and MMP9 were downregulated in tumor tissues from intracranial xenografts with TNC knockdown (magnification: 400×; scale bar=50μm). (**p*<0.05, ***p*<0.01, ****p*<0.001)

**Supplementary Table 1. The ratio of positive Tenascin-c staining in the glioma tissue microarray**

| WHO | Ratio (%) |
| --- | --- |
| I | (1/12) 8.3 |
| II | (18/81) 22 |
| III | (32/72) 44 |
| IV | (35/64) 54.7 |

**Supplementary Table 2.** A list of the human qRT-PCR primers utilized for this study. TNC, Tenascin-c; MMP2, Matrix Metalloproteinase-2; MMP9, Matrix Metalloproteinase-9; FAK, Focal adhesion kinase; VEGFR2, VEGF receptor-2; EPHB2, EPH Receptor B2; YAP, Yes Associated Protein 1; NOTCH1, Notch Receptor 1; GAPDH, Glyceraldehyde-3-Phosphate Dehydrogenase; F, forward; R, reverse.

| TNC | F | TGGAATAAGCAAAGGAAGGCG |
| --- | --- | --- |
| TNC | R | ATGGAGGGTGTACCTCCTGT |
| MMP2 | F | ACCCATTTACACCTACACCAAG |
| MMP2 | R | TGTTTGCAGATCTCAGGAGTG |
| MMP9 | F | CGAACTTTGACAGCGACAAG |
| MMP9 | R | CACTGAGGAATGATCTAAGCCC |
| FAK | F | AAATACGGCGATCATACTGGG |
| FAK | R | TTGGCCTTGACAGAATCCAG |
| VEGFR2 | F | ATAGAAGGTGCCCAGGAAAAG |
| VEGFR2 | R | GTCTTCAGTTCCCCTCCATTG |
| EPHB2 | F | TATGCAGAACTGCGATTTCCAA |
| EBHP2 | R | TGGGTATAGTACCAGTCCTTGTC |
| YAP | F | TAGCCCTGCGTAGCCAGTTA |
| YAP | R | TCATGCTTAGTCCACTGTCTGT |
| NOTCH1 | F | TGCCTGGACAAGATCAATGAG |
| NOTCH1 | R | CAGGTGTAAGTGTTGGGTCC |
| GAPDH | F | ACCACAGTCCATGCCATCAC |
| GAPDH | R | CACCACCCTGTTGCTGTAGCC |
