# Supplementary figures and images for: Tenascin-c mediated vasculogenic mimicry formation via regulation of MMP2/MMP9 in glioma
Source: Cell Death Dis. 2019 Nov 21;10(12):879. doi: 10.1038/s41419-019-2102-3 (PMC6872754; doi:10.1038/s41419-019-2102-3)

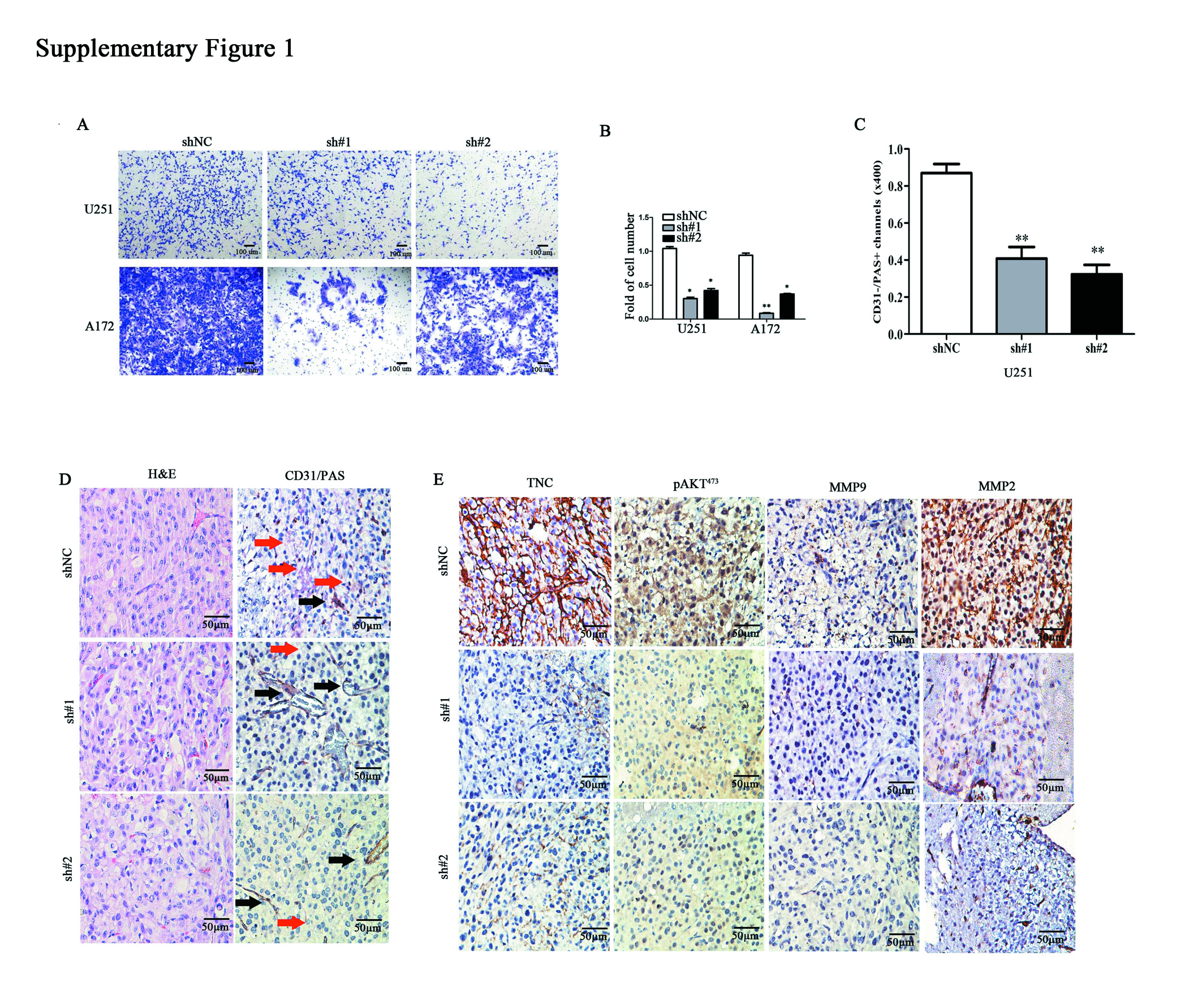

Supplement: Supplementary file 2 — Supplementary Figure 1 [file 41419_2019_2102_MOESM2_ESM.tif]
